# Insecure attachment is associated with math anxiety in middle childhood

**DOI:** 10.3389/fpsyg.2015.01596

**Published:** 2015-10-15

**Authors:** Guy Bosmans, Bert De Smedt

**Affiliations:** Parenting and Special Education Research Group, Faculty of Psychology and Educational Sciences, University of Leuven, LeuvenBelgium

**Keywords:** mathematics achievement, insecure attachment, math anxiety, mediation

## Abstract

Children’s anxiety for situations requiring mathematical problem solving, a concept referred to as math anxiety, has a unique and detrimental impact on concurrent and long-term mathematics achievement and life success. Little is known about the factors that contribute to the emergence of math anxiety. The current study builds on the hypothesis that math anxiety might reflect a maladaptive affect regulation mechanism that is characteristic for insecure attachment relationships. To test this hypothesis, 87 children primary school children (*M*_age_ = 10.34 years; *SD*_age_ = 0.63) filled out questionnaires measuring insecure attachment and math anxiety. They all completed a timed and untimed standardized test of mathematics achievement. Our data revealed that individual differences in math anxiety were significantly related to insecure attachment, independent of age, sex, and IQ. Both tests of mathematics achievement were associated with insecure attachment and this effect was mediated by math anxiety. This study is the first to indicate that math anxiety might develop in the context of insecure parent–child attachment relationships.

## Introduction

There is growing consensus that individual differences in mathematics achievement are not merely a product of cognitive factors, such as numerical magnitude processing (e.g., De Smedt et al., 2013) or working memory (e.g., Friso-Van den Bos et al., 2013), but that such differences are also partially explained by the anxiety to perform tasks involving mathematical problem solving, a concept that has been referred to as math anxiety (e.g., Ma, 1999; Ashcraft et al., 2007; Maloney and Beilock, 2012; Young et al., 2012). Accumulating evidence suggests that from middle childhood onward, some individuals are more prone to develop this math anxiety, which has been defined as “feelings of tension and anxiety that interfere with the manipulation of numbers and the solving of mathematical problems in a wide variety of ordinary life and academic situations” (Richardson and Suinn, 1972, p. 551). Because math anxiety not only impacts on mathematical skills, but also has adverse effects on career choice, employment, and professional success (e.g., Ma, 1999), especially in view of our numerate western society, research is needed to better understand the characteristics of individuals with elevated levels of math anxiety in order to develop appropriate interventions. Against the background of research on broader anxiety-related problems, which has convincingly shown that such problems result from maladaptive coping strategies that some children develop in the context of insecure attachment relationships (Brumariu and Kerns, 2010; Vasey et al., 2014), the present study investigated for the first time the hypothesis that individual differences in math anxiety reflect differences in attachment security.

Various studies have shown that math anxiety has a negative impact on mathematics achievement (e.g., Ashcraft et al., 2007). More specifically, math anxiety disrupts general cognitive capacities, such as working memory resources (e.g., Ashcraft and Kirk, 2001), as well as specific numerical processing skills, such as symbolic magnitude processing (Maloney et al., 2011) and counting (Maloney et al., 2010), that are needed for successful mathematics performance. On the other hand, it leads to the avoidance of mathematics (e.g., Lyons and Beilock, 2012a,b; Maloney and Beilock, 2012). This is nicely illustrated by recent neuroimaging data which indicate that in high math anxious individuals, even the anticipation of performing a mathematical task elicits an increased neural response in brain regions related to visceral threat detection and the experience of pain, including bilateral posterior insula (Lyons and Beilock, 2012a), as well as regions related to cognitive control and the processing of negative emotions, such as the bilateral inferior frontal junction (Lyons and Beilock, 2012b).

While most of the existing body of evidence has focused on adolescents and college students, pointing to a moderate association (*r* between -0.27 and -0.31*)* between math anxiety and mathematical performance (for meta-analyses see Hembree, 1990, *r* = -0.27; Ma, 1999, *r* = -0.31), there is an emergent, yet limited, number of studies that is examining the effect of math anxiety on mathematical performance in primary school children. These data converge to the conclusion that already at this young age elevated levels of math anxiety are associated with poorer mathematics achievement (e.g., Devine et al., 2012; Wu et al., 2012; Young et al., 2012) and that increased levels of mathematical anxiety in second grade coincided with lower gains in children’s mathematics achievement from second to third grade (Vukovic et al., 2013). This association between math anxiety and mathematics achievement is not explained by trait anxiety (Wu et al., 2012) or by test anxiety (Devine et al., 2012). Recent neuroimaging data are beginning to shed light on the neural underpinnings of math anxiety in children (Young et al., 2012). These authors showed that in 7–9-years-old heightened levels of math anxiety are related to increased activity in regions of the right amygdala that are associated with the processing of negative emotions and to decreased activity in posterior parietal and dorsolateral prefrontal cortex networks that are typically recruited during mathematical reasoning.

Although studies that aim to understand individual differences in math anxiety in young children are emerging slowly, research on the origin of broader anxiety-related problems has a much more established tradition (Bernstein et al., 1996). These data have convincingly shown that anxiety-related problems result from the maladaptive coping strategies that some children develop in the context of insecure attachment relationships (Brumariu and Kerns, 2010; Vasey et al., 2014). Bowlby (1969) proposed that the attachment system and the anxiety system are intrinsically interwoven. To promote survival, threat activates the attachment system directing children’s motivational focus toward proximity and support seeking (Cassidy, 2008). Depending on whether or not parents are subsequently experienced as providing responsive support, children will develop either secure or insecure attachment expectations regarding mother’s future availability as a source for support (Bowlby, 1969). Research suggests that insecure attachment fundamentally alters children’s ability to cope with distress, explaining links between attachment and anxiety-related problems (Brumariu et al., 2012). While securely attached distressed children easily seek parental support, insecurely attached distressed children cannot confidently rely on their parents. Hence, they rely on less adaptive, secondary attachment-related coping strategies (Mikulincer and Shaver, 2007; Brenning et al., 2011a). These latter strategies depend on children’s predominant insecure attachment style. More anxiously attached children continue seeking parental support, but their fear that parents will not support them hyper-activates negative emotions, which makes them hyper-vigilant for sources of distress. More avoidantly attached children distance themselves from parents. They no longer seek support, but they deactivate or suppress all emotions and behaviorally avoid sources of distress. In line with the assumption that both maladaptive emotion regulation strategies put children at risk to develop anxiety problems (Vasey et al., 2014), attachment anxiety and attachment avoidance have both been linked with the emergence of anxiety problems (Bar-Haim et al., 2007; Groh et al., 2012).

The hypothesis that math anxiety might be determined by insecure attachment could provide an important link to explain intriguing previous research findings, which demonstrate that insecure attachment predicts poor mathematics achievement (e.g., Keller et al., 2008). To date, attachment theory has failed to provide a strong explanation of this effect, but math anxiety might represent one of the underlying mechanisms. More specifically, it seems reasonable to assume that more anxiously attached children make more mistakes during tasks that involve mathematical problem solving, because their elevated levels of math anxiety during these tasks consume their working memory resources they need to successfully complete these tasks (Mattarella-Micke et al., 2011). Additionally, it is reasonable to assume that more avoidantly attached children might avoid seeking help of parents’ and/or teachers’ while studying, which, in turn, might lead to less proficiency in academic domain knowledge and increased anxiety when they have to perform in that academic domain. This leads to the prediction that the link between insecure attachment and poor mathematics achievement might be mediated by math anxiety.

### The Present Study

The present study is the first to test the hypothesis that individual differences in math anxiety are explained by insecure attachment. We verified whether increased levels of math anxiety were associated with insecure attachment. In addition, we investigated whether math anxiety mediated the previously observed association between insecure attachment and poor mathematics achievement.

The abovementioned hypotheses were tested in a randomly selected sample of fifth graders. We focused on fifth grade because middle childhood is the age period during which math anxiety emerges (e.g., Ma, 1999) and research at this age might have more power to identify associated risk factors. On the other hand, an increasing number of attachment-related studies emphasize that studying attachment in this age-group is essential to understand long-term development of anxiety problems (Bosmans et al., 2014).

Although both parents are equally important attachment figures to understand links between attachment and mathematics achievement (Keller et al., 2008), the current study only focused on attachment to the mother, in order to limit the number of questionnaires that had to be completed by the (young) participants. Mathematics achievement was investigated by means of timed as well as untimed standardized mathematics tests. To evaluate whether the associations between insecure attachment, math anxiety and mathematics achievement were not explained by general intellectual abilities, a measure of intelligence was administered.

## Materials and Methods

### Participants

Participants were 87 children (46 girls) that were randomly selected from three Flemish primary schools. They all were attending fifth grade and their mean age was 10.34 years (*SD* = 0.63). The children came from a variety of socioeconomic backgrounds. The parents of 15 children were divorced and 4 children had a deceased father. Eighty-five children filled out the attachment questionnaires about their biological mother, two children reported on their relationship with their stepmother.

### Procedure

Parents were informed about the study via letters distributed in the classroom, and they all gave consent for participation. Children were tested at their own schools during school hours. All measures were group administered.

### Measures

#### Attachment

Children completed an adapted version of the Experiences in Close Relationships Scale-Revised (ECR-R; Fraley et al., 2000, adapted for children as the ECR-RC by Brenning et al., 2011b). The ECR-RC assessed the two dimensions central in attachment-related affect regulation: attachment anxiety and avoidance in relationship to the mother. Attachment anxiety was measured with 18 items tapping into feelings of fear of abandonment and strong desires for interpersonal merger (e.g., “I worry about being abandoned by my mother”). Attachment avoidance was assessed with 18 items tapping into discomfort with closeness, dependence, and intimate self-disclosure (e.g., “I prefer not to show to my mother how I feel deep down”). Items were rated on a seven-point Likert scale ranging from not at all ( = 1) to very much ( = 7). Both subscales have strong internal consistency and validity (Brenning et al., 2011b). Cronbach’s αs of the ECR-RC in this study were 0.90 and 0.80 for attachment anxiety and avoidance, respectively.

#### Math Anxiety

The Mathematics Anxiety Rating Scale for Adolescents (MARS-A; Suinn and Edwards, 1982) was adapted to use with primary school children. The original questionnaire was reduced to 15 items that described mathematical situations with which children are often confronted with (e.g., “How nervous are you when you are called during math class”). Children were asked to indicate on a five-point Likert scale how anxious they were in these situations, ranging from not at all ( = 1) to very anxious ( = 5). This test had a high internal consistency in the current sample, i.e., Cronbach α = 0.88.

#### Mathematics Achievement

Two standardized mathematics achievement tests were administered. The Tempo Test Arithmetic (de Vos, 1992) was used as a timed measure of mathematics achievement. In this test, children had to solve basic arithmetic combinations (e.g., 6 + 5) of increasing difficulty as fast and accurately as possible. The test consisted of five columns of 40 items, comprising additions, subtractions, multiplications, divisions, and mixed problems. For each column, children had to solve as many problems as possible within 1 min. The total number of correctly solved problems across all columns was used in all subsequent analyses. We also administered the untimed curriculum-based standardized achievement test of mathematics (Dudal, 2000). This test consisted of 60 items that covered a wide range of mathematical skills, such as number knowledge, calculation, word problem solving, measurement, and geometry. The total number of correctly solved items was used in all subsequent analyses.

#### Intelligence

Raven’s Progressive Matrices (Raven et al., 1992) was used as a measure of children’s intellectual ability. The test consists of 60 items and raw scores were used in all analyses.

## Results

### Preliminary Analyses

Descriptive statistics of the variables under study are reported in **Table 1**. All variables were well-distributed with no floor or ceiling effects. There were no sex differences (*p*s: 0.13–0.97), except for the timed mathematics achievement test [*F*(1,85) = 4.48, *p* = 0.037] on which girls had lower scores (*M* = 71.02, *SD* = 11.60) than boys (*M* = 76.12, *SD* = 10.76).

**Table 1:** Correlation and descriptive statistics (*n* = 87).

	1	2	3	4	5	6
(1) Math anxiety	–					
(2) Attachment anxiety	0.25^∗^	–				
(3) Attachment avoidance	0.27^∗^	0.54^∗∗∗^	–			
(4) Timed math	-0.42^∗∗∗^	-0.18^†^	-0.01	–		
(5) Untimed math	-0.43^∗∗∗^	-0.31^∗^	-0.16	0.46^∗∗∗^	–	
(6) IQ	-0.43^∗∗∗^	-0.14	-0.09	0.26^∗^	0.58^∗∗∗^	–
*M*	25.30	2.28	2.54	73.43	41.43	42.47
*SD*	7.80	0.98	0.87	11.44	10.07	5.98
Minimum	15	1.00	1.00	46	14	24
Maximum	48	5.00	4.72	92	57	55
Maximum possible	75	7.00	7.00	120	60	60


^†^*p* < 0.1; ^∗^*p* < 0.05; ^∗∗∗^*p* < 0.001.

### Math Anxiety and Insecure Attachment

Both measures of insecure attachment were significantly related to mathematics anxiety (**Table 1**), indicating that children with less secure attachment showed higher levels of mathematics anxiety. We next calculated partial correlations with age, sex, and IQ as control variables to verify whether these associations were affected by age, sex, or intelligence. When controlling for age, the associations between insecure attachment and mathematics anxiety remained significant (attachment anxiety: *r_p_* = 0.21, *p* = 0.048; attachment avoidance *r_p_* = 0.24, *p* = 0.023). Similarly, these associations remained significant when sex (attachment anxiety: *r_p_* = 0.24, *p* = 0.029; attachment avoidance: *r_p_* = 0.27, *p* = 0.012) and IQ (attachment anxiety: *r_p_* = 0.22, *p* = 0.048; attachment avoidance: *r_p_* = 0.25, *p* = 0.019) were taken into account.

### Insecure Attachment, Math Anxiety, and Mathematics Achievement

The correlations in **Table 1** showed the expected negative association between math anxiety and timed as well as untimed measures of mathematics achievement. On the other hand, attachment anxiety correlated with mathematics achievement, in particular with the untimed test. In the next mediation analyses, we explored whether math anxiety mediated the association between insecure attachment and poor mathematics achievement.

We tested this mediation hypothesis by following recommendations by MacKinnon et al. (2004), with a non-parametric resampling bias-corrected bootstrap approach with 10.000 resamples drawn with replacement from the original sample (*n* = 87) to derive the confidence interval (CI) for the unstandardized regression coefficient of the indirect effect (Hayes, 2009). The indirect effect through math anxiety was considered as significant when 0 was not part of the CI. To test the significance of the indirect effect, we performed four mediation analyses using Model 4 of the PROCESS Macro provided by Hayes (2013) with attachment as predictor, math anxiety as mediator, and the two mathematics achievement measures as criterion variables. If the indirect effect is significant, mediation has occurred. To control for effects of age and sex, all mediation analyses were carried out with these variables as control variables.

When predicting *timed mathematical achievement*, the initial marginally significant association with attachment anxiety was reduced to non-significance after taking into account the effect of Math Anxiety (β = -0.07, *p* = 0.54). Zero was not part of the 95% CI around the indirect effect (**Table 2**), suggesting that math anxiety mediated the link between attachment anxiety and timed mathematical achievement. The entire model explained 20% (*p <* 0.001) of the variance in timed mathematical achievement. Even though there was no direct effect of attachment avoidance on timed mathematical achievement, there is accumulating evidence, which suggests that absent direct effects could be the result of significant indirect effects; this leads to the recommendation to test for indirect effects in spite of absent direct effects (Rucker et al., 2011). Confirming the accumulating evidence, and in line with our expectations, the indirect effect of math anxiety on the association between attachment avoidance and timed mathematical achievement was significant (**Table 2**). The entire model explained 21% (*p* < 0.001) of the variance.

**Table 2:** Confidence intervals around unstandardized regression coefficients for indirect effects.

	Timed math		Untimed math	
	95% CI indirect effect		95% CI indirect effect	
Attachment anxiety	-2.15 <	< -0.11	-1.79 <	< -0.02
Attachment avoidance	-2.92 <	< -0.31	-2.24 <	< -0.18


When predicting *untimed mathematical achievement*, the initial significant association with attachment anxiety, was reduced, but remained significant after taking into account the effect of math anxiety (β = -0.22, *p* = 0.03). The indirect effect was significant (**Table 2**) suggesting that math anxiety mediates the link between attachment anxiety and untimed mathematical achievement. The entire model explained 23% (*p* < 0.001) of the variance in untimed mathematical achievement. Although the initial direct effect of attachment avoidance on untimed mathematical achievement was not significant (β = -0.05, *p* = 0.65) after taking into account math anxiety, the indirect effect was significant (**Table 2**) and the entire model explained 19% (*p* < 0.001) of the variance in untimed mathematical achievement.

As a final step, we evaluated whether the mediation effects remained when children’s intelligence was taken into account as a control variable. The mediation effects were largely unaffected by intelligence, except for the indirect effect of Attachment Anxiety on Untimed Mathematical Achievement, which was slightly suppressed, but remained marginally significant (90% CI: -1.15 < *b* < -0.05).

## Discussion

There is an increasingly emerging literature that stresses the importance of affect regulation mechanisms in individual differences in academic competence. In the context of mathematics achievement, research has pointed to the detrimental role of mathematics anxiety on mathematical performance (e.g., Hembree, 1990; Ma, 1999; Ashcraft et al., 2007). The current study is the first to investigate whether children’s emerging mathematical anxiety could be related to insecure attachment, and whether mathematical anxiety explains the associations between insecure attachment and mathematics achievement. Our data suggest that higher levels of mathematics anxiety are associated with insecure attachment and that mathematical anxiety mediates the association between insecure attachment and mathematical achievement. In other words, these data indicate that less securely attached children are more likely to show math anxiety, and therefore are more vulnerable to perform poorly on mathematical tasks. As such, these data highlight the importance of considering children’s insecure attachment when studying the origins of math anxiety.

In line with the existing body of evidence, the current study observed and replicated the significant association between mathematics anxiety and mathematical achievement in primary school children (Devine et al., 2012; Wu et al., 2012; Young et al., 2012; Vukovic et al., 2013). It is interesting to note that this association was observed for timed as well as untimed standardized tests of mathematical performance, which suggests that mathematics anxiety is linked to both time-pressured and non-pressured testing situations.

Individual differences in mathematical anxiety were significantly related to insecure attachment. These associations were independent of age, sex, and IQ. The current data are the first to indicate that insecure attachment toward the mother might be an important contextual factor related to children’s math anxiety. This finding suggests that adverse social factors could contribute to individual differences in children’s mathematical anxiety. Unfortunately, the current study’s cross-sectional research design does not allow us to draw conclusions about the time course of these associations. It remains to be seen whether insecurely attached children are at risk to develop math anxiety, or vice versa, or that the association between these two non-cognitive factors is bidirectional. This all motivates longitudinal research in which attachment is investigated as a possible predictor of subsequent math anxiety development.

Math anxiety mediated the association between attachment and mathematical performance. This indicates that math anxiety might be an underlying mechanism for the previously observed associations between insecure attachment and poor mathematics achievement (e.g., Keller et al., 2008). It might be contended that less securely attached children make more errors during mathematical problem solving due to their elevated levels of math anxiety. It also might be that children who choose to not seek help from parents or teachers, due to insecure attachment, would be less likely to receive help in mathematics from parents or teachers, thus leading to less proficient domain knowledge. Future studies should explore these possibilities more directly.

The observed pattern of associations appeared to be specific and was not explained by recourse to intelligence. Also, findings were similar for both timed and untimed measures of mathematics achievement, which indicates that insecurely attached children’s levels of math anxiety increases for mathematics in general and not only when they have to perform under time pressure. The interrelations between insecure attachment and math anxiety accounted for 18–23% of the variance in children’s mathematical performance and point to an important role of these non-cognitive factors in explaining individual differences in mathematics achievement, yet future studies should explore how these non-cognitive factors interact with other well-known cognitive predictors of mathematics achievement, such as numerical magnitude processing (e.g., De Smedt et al., 2013) or working memory (e.g., Friso-Van den Bos et al., 2013).

The current findings provide the first indication that attachment-related factors are important to understand math anxiety, yet some limitations need to be taken into account when evaluating the conclusions of the present study. One limitation deals with our use of self-reported attachment and math anxiety measures and this might have affected our findings. Attachment researchers have traditionally raised concerns regarding the validity of self-reported attachment (Ainsworth, 1985). More specifically, self-report is considered vulnerable for underreporting insecure attachment, and narrative measures, such as attachment interviews are generally considered to be more appropriate because they are less vulnerable to defensive response styles (e.g., Main et al., 1985). However, psychometric research on middle childhood attachment increasingly shows that these concerns are not applicable to this age-group, as these children appear to respond similarly to self-report and narrative measures (e.g., Psouni and Apetroaia, 2014) and both measurement approaches are equally valid indicators of adverse developmental outcomes (Kerns et al., 2007). Relatedly, self-reported anxiety problems are also vulnerable to defensive underreport, yet validation research has demonstrated that self-report is the most valid strategy to identify anxiety-related problems (e.g., Achenbach et al., 1987). One important avenue for future research might be to combine self-reports with real-time physiological measures of stress and anxiety. Such approach has been successfully applied in studying the association between anxiety and attachment (e.g., Gilissen et al., 2008), and it therefore might be particularly useful to collect such physiological data immediately before or during the execution of mathematical problem solving tasks.

The current study only focused on attachment toward mother. The question remains whether the similar effects are observed for other attachment figures like fathers or grandparents. Future research should therefore include the role of attachment to fathers too, because research suggests that a good relationship with father can buffer the negative effect of mother on children’s anxiety development (e.g., Bögels and Phares, 2008).

An important limitation of the current study was that it did not include measures of trait anxiety and general anxiety. It indeed cannot be excluded that the association between attachment and math anxiety only reflects that trait anxious children are both more likely to be insecurely attached and to display math anxiety problems. Against the background of previous research, such an explanation seems less likely. Specifically, attachment research has convincingly shown that differences in attachment security are not linked to temperament (Vaughn et al., 2008). Similarly, research suggests that math anxiety and trait anxiety are different constructs (Devine et al., 2012; Wu et al., 2012). While this suggests that controlling for trait anxiety would not have changed the results of the current study, it remains important to rule out this alternative explanation. On the other hand, it would be interesting to investigate to what extent the observed association between insecure attachment and math anxiety merely reflects a general anxiety effect. It is possible that for insecurely attached children math anxiety is part of a broader anxiety problem. For example, if an insecurely attached child has difficulties with math or is exposed to a high degree of negative attitudes about math, it might develop math anxiety. This math anxiety could lead to lower math achievement through either transient reductions in working memory or the avoidance of mathematics. These possibilities should be explored in future studies. Our findings have important implications for the development of new intervention strategies. Current practices to address math anxiety include not only techniques that are used to overcome other types of anxiety, such as desensitization (e.g., Brunyé et al., 2013), but also more specific methods that aim to address the origins of math anxiety, such as the improvement of math learning experiences (e.g., Kramarski et al., 2010). In view of the current data that children with insecure attachment are vulnerable to the development of math anxiety, and consequently to poor math performance, interventions that are tailored to insecure attachment-related mechanisms might be particularly useful. For example, research on teacher–child relationships in insecurely attached children has shown that teacher sensitivity can buffer against the maladaptive effects of children’s insecure attachment (Buyse et al., 2011). It is therefore likely that improving teachers’ skills to respond sensitively to children might decrease insecurely attached children’s math anxiety and increase their mathematics achievement.

## Conflict of Interest Statement

The authors declare that the research was conducted in the absence of any commercial or financial relationships that could be construed as a potential conflict of interest.
